# Foreign Summary

**Published:** 1839-01-05

**Authors:** 


### FOREIGN SUMMARY.

*Case of Rupture of the Inferior Vena Cava.*—A man, fifty years of age, had suffered, in youth, from repeated attacks of hemoptysis. For the last eighteen years he had also been afflicted with hemorrhoids, and a varicose state of the veins of the foot, which had gradually advanced to the thigh and groin. Three or four weeks before his death, the patient had been ill with a slow intermittent fever, from which he was beginning to recover, when, four days before his decease, he was suddenly seized with pains in the breast, accompanied with oppression, vomiting, and diarrhœa. These symptoms gradually subsided, and the patient seemed convalescent, when he had a relapse. He was attacked with violent pain in the right lumbar region; his respiration was excessively oppressed, and he was incessantly complaining of want of air. Convulsions followed, and death took place at the end of four hours.

Some traces of chronic inflammation were found in the lungs. The right auricle of the heart was considerably enlarged, while its parietes were much thinner than in the normal state. The right ventricle was also much thinner than usual. The pericardium contained two or three spoonsful of pure serum. The liver was very black, soft, and congested with blood, the vena portarum was dilated, and the spleen twice its ordinary size. The whole abdominal venous system was in a state of remarkable dilatation. The right lumbar and inguinal regions contained a quantity of coagulated blood. By careful examination the course of the hæmorrhage was traced to the inferior vena cava, near its junction with the iliac vein. This portion of the vessel was remarkably dilated, and presented several openings of the size of a pin's head. The parietes of the vein did not appear abnormally thin, although the dilatation occupied its entire calibre.—*Berlin Medicin. Zeitung.*

*Diosma Crenata—The Buchu.*—The natives of the Cape of Good Hope ascribe the most extraordinary virtues to the leaves of the family of the diosmeæ, and to some species of plants which approach to it in general characteristics. Botanists have subdivided this genus, and the buchu now belongs to one of those subdivisions called barosma. It is owing to the perseverance and unwearied attention of the late Dr. Reece that we are now familiar with the intrinsic merits of this medicine, and to the earnestness with which he inculcated his own views must be ascribed its introduction into practice. From him I gathered all the information I possess upon this plant, and likewise a vast deal of useful knowledge of the value of some of those of which I have already spoken. He wrote a little pamphlet, in the year 1825, which embraces some very favourable reports of different authors and physicians who had recommended this drug; he was not too sanguine in his estimation, and I have had every reason to be satisfied with the trials I have made of the preparations he employed. It is a shrub which flowers at the Cape of Good Hope throughout August, September, and October; it is perennial, erect; it rises to the height of about two feet; the branches have somewhat of a purplish colour; the leaves opposite, ovate, crenate, of a dark-green colour in the upper lamina, somewhat paler on the lower; they are full of small transparent punctures, particularly at the edge. The flowers are solitary, on short pedicles, white, or of a pale reddish hue; the calyx consists of five deep ovate segments, the corolla of five elliptic oblong segments, slightly spreading; the nectaries are five linear lanceolate scales, crowning the germen, the filaments are five, awl-shaped, bearing ovate incumbent anthers; the germen is superior, the style erect, the length of the stamens, with a simple stigma; the capsule is ovate, containing an oblong solitary seed, inclosed in an elastic coat. The leaves, as they are imported into this country, have a very agreeable aromatic odour, by which they are distinguished from senna, to which they bear a very striking resemblance; the short petiole which remains attached to them is channelled, the upper surface smooth, and has a yellowish-olive hue; the under surface is somewhat paler, and is studded with large glands, having each of them an excretory pore. These afford, on distillation, an essential oil, resembling a mixture of oil of rue, juniper, and camphor; this odour has been imitated, and Dr. Reece states, that a species of senna imported from the Cape was artificially prepared by a mixture of this kind; the extractive matter is slightly bitter and mucilaginous. The virtues of the leaves are imparted to boiling water by infusion, and also to proof spirit. The saturated tincture and the extract, when carefully prepared, so as to retain the volatile parts, are the best preparations. M. Cadet has made an analysis of the leaves, and in the third volume of the "Journal de Chimie" it is given to us, and we learn from it that they contain essential oil, gum, extractive matter, chlorophylle, acid, resin. The London and Dublin Pharmacopœias have both received infusions into their formulas; that of the former is thus made:—Take of buchu an ounce, boiling distilled water a pint; macerate for four hours in a lightly covered vessel, and strain. An ounce or two ounces may be given in those cases in which this drug is required; but a tincture is a very useful preparation: that of the Dublin College is formed of two ounces of the buchu leaves to a pint of proof spirit, and maceration is to go forward for seven days. Dr. Reece employed a tincture much more abounding with the volatile oil, and a very excellent medicine I have always found it. He imitated a preparation made by the natives at the Cape, and highly approved of by the Dutch, which they call buchu brandy; it is formed by distilling the leaves in the dregs of wine, and in this state they consider it as a sovereign remedy for all chronic disorders, and even for acute ones of the stomach and bladder. Thunberg observes that the Hottentots reduce the leaves of a plant, which he describes "uniflora, pulchella, crenata et betulina cognomine buchu," and this is done by pounding them by stones; they besmear the body with this, made up with fatty matter, whence they have a disagreeable and very remarkable odour. Burchell, in his "Travels in Africa," likewise mentions the uses to which the Hottentots applied the leaves of this plant, more especially noticing that they made an application of an infusion to recent wounds. In Holland these leaves were introduced medicinally much earlier than they were in this country, and their employment received the sanction of high medical authority.

In the different varieties of mucous discharge from the urethra, whether they be the result of long-continued excess, or of momentary irritation, the leaves of the buchu, either in the form of infusion, or of tincture, afford the most certain prospect of recovery. Persons of relaxed habits, in whom a collection of mucous not unfrequently occurs in the bulbous part of the urethra from the prostate gland and the seminal vessels, which is mistaken for the generative secretion, and those who, in early life, are subject to a collection of clear transparent mucus in the urethra, will find this medicine completely arrest the progress of these disordered states. One of the volumes of the "Dublin Medical Transactions," contains a communication from Dr. M'Dowall on the beneficial effects of the buchu leaves in cases of irritative affections and chronic inflammation of the bladder, urethra, &c. The doctor observes, that the chronic form of inflammation very frequently affects the mucous membrane of the bladder, and, when neglected, extends up the ureters to the kidneys, producing a degree of local irritation sufficient to disturb the general health. These complaints may, in general, be traced to mismanaged gonorrhœa, retention of urine, disease of the prostate gland, stricture of the urethra, the action of calculi, and the rude use of the bougie. The saturated tincture, and the extract, when carefully prepared, so as to retain the volatile parts, are the best preparations. The dose of the former is two teaspoonsful, and of the latter ten grains.—*Dr. Sigmond's Lecture—Lancet.*

---

*Sugar in the blood.*—The question of the presence of sugar in the blood of diabetic persons, has long been a matter of dispute amongst chemists. It appears certain, however, that Ambrosiani extracted saccharine matter in a state of purity from the serum of diabetic blood, while recent experiments of Mr. M'Grigor lead to the idea that it exists in several of the excretions also. The following process is described by Dr. G. O. Rees, in the last number of "Guy's Hospital Reports:"

"The mass of blood is to be evaporated to dryness, over a water-bath; the dried mass to be comminuted, and digested for several hours in boiling water: the aqueous solution is to be filtered off, evaporated to dryness, and the dried residuum digested in alcohol of sp. gr. 0.825: the alcoholic solution so formed is to be filtered, or carefully poured off, evaporated to dryness, and the dry mass treated several times with rectified ether, which dissolves out urea, and also some fatty matter; leaving behind the sugar, in admixture with osmazome and chloride of sodium: this mass, on being dissolved in alcohol, and the solution allowed to evaporate spontaneously in a flat glass dish, affords mixed crystals of alkaline chloride and diabetic sugar; which are easily distinguishable from each other, and allow of being separated mechanically, by shaking them up in alcohol, when the chloride sinks; and the sugar, being principally collected above, may be removed, for examination, by careful use of the spatula: the alcohol must not, of course, be allowed to remain long in contact with the crystals, as it would re-dissolve them. It is a matter of surprise, that sugar has not been long ago detected in the blood of diabetic patients, though not separated from it; for the alcoholic extract of the serum, when mixed with water, will, after a few days, give off carbonic acid; which, in addition to the sweetish taste, and syrupy smell of the evaporated alcoholic extract, is a sufficient evidence of the presence of sugar. The following is an analysis of 1000 grains of diabetic serum:

| | |
|---|---|
| Water . . . . . . . . | 908.50 |
| Albumen (yielding traces of phosphate of lime and oxide of iron, on incineration) . . . . . . . | 80.35 |
| Fatty matters . . . . . . | 0.95 |
| Diabetic sugar . . . . . . | 1.80 |
| Animal extractive, soluble in alcohol, urea . . . . . . . | 2.20 |
| Albuminate of soda . . . . . | 0.80 |
| Alkaline chloride, with traces of phosphate . . . . . . . } Alkaline carbonate, and trace of sulphate, the results of incineration . } | 4.40 |
| Loss . . . . . . . . | 1.00 |
| *Lancet.*] | 100.000 |

